# Efficacy of an Attachment-Based Intervention Model on Health Indices in Children with Chronic Disease and Their Mothers

**DOI:** 10.1007/s10488-018-0873-y

**Published:** 2018-05-07

**Authors:** Fateme Dehghani-Arani, Mohammad Ali Besharat, Victoria A. Fitton, Asghar Aghamohammadi

**Affiliations:** 1 Department of Psychology, University of Tehran, Dr Kardan Street, Nasr Bridge, Jalal Al-Ahmad Street, Chamran Highway, Tehran, 1445983861 Iran; 2 School of Social Work, Michigan State University, 242 Baker Hall, 655 Auditorium Road, East Lansing, MI 48824 USA; 3 Pediatric Medical Center Hospital, Tehran University of Medical Science, Dr Gharib Street, Tehran, Iran

**Keywords:** Chronic disease, Attachment, Health indices, Mother–child dyads, Attachment-based intervention

## Abstract

Studies have shown significant relationship between health conditions and attachment. This study aimed to examine an attachment-based intervention model named mother–child-disease triangle (MCDT) on health indices in children with chronic disease and their mothers. This randomized trial study included 22 volunteer children aged 12–18 years undergoing medical treatment for a chronic disease and their mothers. After evaluation by 28-form General Health Questionnaire (GHQ-28), inventory of parent and peer attachment (IPPA), 28-form Child Health Questionnaire (CHQ-28) and Illness Perception Questionnaire (IPQ), the mother–child dyads were paired on the basis of IPPA scores. These pairs were then randomly assigned to an experimental or control group. The experimental group received ten 90-min sessions of MCDT over a 7-week period. Meanwhile, the control group received ten simple conversational sessions as a dummy intervention. In accordance with this study’s pre-test/post-test design, both groups were evaluated once again after completing their respective treatment. Multivariate analysis of covariance (MANCOVA) showed members of the experimental group to have significantly stronger attachment and better physiological and psychosocial health than those in the control group. These findings suggest that attachment-based interventions can be used to improve the effectiveness of treatment among children with chronic disease and their mothers.

## Introduction

Health psychologists are beginning to apply attachment theory in the treatment of chronic medical conditions with children because of the powerful protective factors offered in a secure maternal-child bond/attachment (Pietromonaco and Powers 2015). The term “maternal” is used here in the attachment description because the vast majority of early attachment relationships are formed with the mother, but attachment relationships form with the primary caregiver regardless of the person or relationship. An attachment system develops in the infant from that early significant bond to the mother or primary caregiver. In order to understand the appeal of this additional research factor in child medical conditions, a brief description of attachment theory follows.

Attachment theory is a perspective on the way close relationships provide a secure base for infants and children that has lifelong implications on all domains of life (Ainsworth 1967, 1969; Bowlby 1958, 1988). Attachment theory assumes that maternal sensitivity, responsiveness, and attunement are central components in the quality of a child’s attachment to her/his mother (Ainsworth 1969; Ainsworth et al. 1978). Attachment and secure base functions operate to promote child and personality development, affect and behavior regulation, cognitive development, protective factors for physical wellbeing, psychic safety, and set the stage for all future human relationships (Fitton 2012). Attachment theory presupposes evolutionary biological necessity. Attachment behaviors must exist and be reciprocated for the infant to survive both physically and psychically (Bowlby 1958). The bond serves to protect the infant/child from fear and harm and sets the stage for the formation of the caregiver as the secure base thereby offering the developing child a safe place from which to explore the world. The child internalizes early experiences with her/his mother and forms enduring cognitive core beliefs or “working models” about the self, others and the world (Bowlby 1980; Bretherton 1985). Relational attunement and developmental assistance are key features of attachment relationships. These are also key features in mother–child experiences when living with and working through chronic disease situations for children.

Chronic disease is a distress situation (Kidd et al. 2016; Maunder and Hunter 2009). From an attachment theory perspective, physical and/or psychological distress activates the attachment system signaling the mother assistance is needed by the child (Bowlby 1958). This theoretical frame aligns with the current medical treatment model, which includes and encourages parents in the visitation and treatment process. This is a lasting legacy of Bowlby and Robertson (1952). Their film and report, *a 2-year-old goes to hospital*, revolutionized the Western hospital system in the 1950s which previously restricted parental visitation and thereby created needless attachment disruption in child-parent relationships. Therefore, if physical distress activates a child’s attachment seeking behavior, it only makes sense to include attachment-based therapy models in the treatment of children with chronic illnesses.

Although studying attachment in relation to disease/health is a relatively recent phenomenon (Maunder and Hunter 2004), some health studies demonstrate a link between secure attachment and mechanisms related to physical health and positive outcomes of chronic diseases (Favez et al. 2017; Kidd et al. 2016; Agostini et al. 2014; Calvo et al. 2014;; Robles and Kane 2014;; van der Meer 2015). These studies do not address causality but suggest attachment style affects physical health through its impact on symptom amplification, health behavior, stress response, patient-provider relationships and social support utilization. According to the above studies, in a medical situation, individuals with a secure attachment style moderately rely on attachment figures for safety and trust to mediate the impact of the disease. However, individuals with an anxious attachment, have a hyperactive attachment system and develop an extreme dependency on others in order to obtain proximity and social support. Overt expressions of distress and poor affect regulation in these individuals are seen as efforts to engage treating professionals for comfort and security. Conversely, avoidant individuals tend to deactivate the attachment system, to suppress their attachment needs and perceived negative emotions and are, therefore, less likely to use social supports or medical staff for stress relief. However, their behaviors, which might include underreporting symptoms and resisting medical interventions, interfere with treatment (also in Gur-Yaish et al. 2016; Klest and Philippon 2016; Cassedy et al. 2015).

Kidd et al. (2016) explain in a new study on patients with coronary artery bypass graft (CABG) surgery, emotional symptoms may be experienced more intensely in patients with attachment anxiety because of the utilization of hypervigilant strategies to identify potential threat. But they report attachment avoidance may be a protective factor for mood during CABG surgery. These researchers finally suggest that attachment style may potentially be an important motivational system involved in coping with disease situations through the appraisal of stress and the patient’s coping resources, ability to rely on others and perception of social support availability. They also mention that attachment anxiety predicts higher levels of interleukin-6, greater sleep disturbance and longer hospital stay. Finally, Favez et al. (2017) demonstrate similar results in women with breast cancer indicating higher attachment anxiety and lower couple satisfaction are both predictive of more criticisms in these subjects. To further explicate the attachment and disease linkage, Maunder and Hunter (2009), Meredith et al. (2008) and Pietromonaco et al. (2013) unanimously hypothesize disease as a stressful situation which activates patients’ attachment styles and related cognitive, emotional, and behavioral characteristics. These characteristics in turn affect the individual’s bio-psychosocial functioning and physical health (Pietromonaco and Powers 2015). This hypothesis is based on Bowlby’s (1988) theory that attachment and stress are related by evolution; the stress response is triggered by environmental threat and the function of the attachment system is to increase security and proximity and to maximize potential safety and health in the face of that environmental threat.

The above review on the effects of attachment on physical health conditions, underlines the necessity of supplementing medical protocols with attachment-based interventions (Kidd et al. 2016; Pietromonaco et al. 2013; Stanton and Campbell 2014). Surprisingly, based on the current literature, there are no clinical and experimental studies on using attachment-based interventions in medical conditions (Alonso et al. 2016; Robles and Kane 2014). Maunder and Hunter (2004) describe a rationale for a clinical integration of attachment theory and existential psychology and sketch a technique of brief attachment-existential psychotherapy directed toward the treatment of medically unexplained symptoms. There are no empirical studies on this proposed therapy but the principles are similar to the rationale for the current study. Pietromonaco et al. (2013) discuss the extensive results of the attachment-disease linkage and necessity of incorporating attachment-based interventions in medical conditions but previous psychological interventions in medical situations do not consider disease as part of the intervention methods. Therefore, this study introduces and preliminarily examines the efficacy of a newly proposed attachment-based intervention model, mother–child-disease triangle (MCDT), which specifically targets children with chronic disease and their mothers.

Justification for the MCDT rather than an approach such as cognitive behavioral therapy or trauma-focused cognitive behavioral therapy is that the framework is designed for flexibility, allowing the treatment specialist to utilize a wide range of intervention models greater than indicated in one specific treatment protocol. For example, MCDT is trauma informed and, therefore, much of the psychoeducational material used in treatment sessions mirrors CBT and TF-CBT—left-brain activity. MCDT also allows for the right brain work of expressive therapies like play and art. And MCDT is grounded in attachment theory with the added advantage of strengthening the mother–child attachment relationship.

## Method

### Participants and Procedure

The immunology, oncology and hemodialysis clinical departments’ doctors of the Tehran Pediatric Medical Center Hospital were asked to introduce the researcher to the bedridden children who had passed the acute phase of their chronic disease and were undergoing medical treatment in the hospital setting. A total of 87 children and their mothers, who had been referred by the doctors, were interviewed and screened. Children and their mothers were not given specific details of the study but kept blind to the process. The sessions were described as a series of psychological visits which could help them adjust to their medical situation. Children 12–18 years old, with a chronic immunology defect or thalassemia or kidney disease and confined to the Tehran Pediatric Medical Center Hospital for a minimum of 3 months, as well as their mothers, met inclusion criteria. All children were secondary or high school level students living with both parents. The mothers’ educational level was at least secondary level. Exclusion criteria included comorbidities such as anoxia, head trauma, stroke, encephalitis or another chronic disease in children. Twenty-two children and their mothers met eligibility criteria and agreed to participate.

After providing signed informed consent, in the pre-treatment stage, all mothers filled out the inventory of parent and peer attachment (IPPA; Armsden and Greenberg 1987), Parenting Stress Index (PSI; Abidin 1983), 28-form General Health Questionnaire (GHQ-28; Goldberg and Hillier 1979) and 28-form Child Health Questionnaire (CHQ-28; Landgraf and Abetz 1996). The children completed the Illness Perception Questionnaire (IPQ; Weinman et al. 1996). The pre-test assessments were completed a week before starting the intervention sessions. Afterward, the mother–child dyads were randomized and assigned in two groups. The two groups were matched on the basis of the children and mothers’ attachment scores. One group was randomly chosen as the experimental group and the other as the control group. The two groups were also equivalent on other demographic characteristics such as the mothers’ age (experimental group: *M* = 40.6, *SD* = 5.2; control group: *M* = 41.6, *SD* = 4.9), child’s age (experimental group: *M* = 15.36, *SD* = 2.15; control group: *M* = 15.4, *SD* = 1.96), family’s number of children (experimental group: *M* = 2.27, *SD* = 1.1; control group: *M* = 2.27, *SD* = .91), child’s sex (both groups included six girls and five boys) and kind of disease (each group included three children with immunology defect, five children with thalassemia, and three children with kidney disease).

The experimental group received ten sessions of MCDT in a consecutive 7-week period. Sessions began after the pre-test assessment. The first week, one session was held and scheduled as the startup and assessment session. The next 3 weeks, two sessions were held each week and scheduled for mother and child to each have individual sessions (each week one session for mother and one session for child). The last 3 weeks, one session was held each week and scheduled for visiting the mother–child dyad. The average length of each session was 1 h for individual sessions and 90 min for mother–child dyad sessions. Simultaneously, the control group received ten simple conversational sessions in which subjects were visited by the same psychotherapist. Routine activities were discussed but no psychotherapeutic interventions were applied. All of the MCDT and comparison sessions were conducted by a doctoral student in clinical health psychology who had been trained in MCDT and simple conversational sessions. The study sessions were supervised by a full professor in clinical psychology, also an expert in the attachment field. At the end of each MCDT and simple conversational session, the therapist reported that session to the supervisor and received comments and viewpoints. All 22 participating mother–child dyads attended every session. One week after the last session, in the post-treatment stage, all the mother–child dyads were again assessed using the same questionnaires. The pre- and post-test assessments were managed by a nurse who was blind regarding the study. All the recruitment procedures and intervention sessions were completed during the children’s inpatient hospital stay at Tehran Pediatric Medical Center Hospital in 10 weeks and supervised by the hospital research review board. The results obtained in the pre- and post-treatment phases for the experimental and control groups were analyzed using the SPSS.20 tool.

### Attachment-Based Intervention Model of Mother–Child-Disease Triangle (MCDT)

MCDT was designed for children aged 10–18 years who have a chronic disease and their mothers. Basic principles from the previously described research and theories form the background of this theoretical model. The first principle of the MCDT model is based on Bowlby’s (1988) theory that to impact attachment in a positive, secure direction, the intervention must simultaneously address the internal working models of both mother and child each derived from their respective early attachment relationships. The second principle is based on Bartholomew and Horowitz’s (1991) theory that attachment is postulated to be the result of mental representations of both self and others that could be positive or negative. The third principle is based on Maunder and Hunter’s (2004) stress-focused model of disease behavior-attachment where disease, as a stressor, triggers the person’s previously developed attachment style. Furthermore, the framework and contents of the MCDT sessions are based on the Meredith et al. (2008) and Sadava et al. (2009) models in which cognitive, emotional, and behavioral states of the patient are the mediating factors in the relationship between attachment style and medical conditions. Also taken into consideration is the Pietromonaco et al. (2013) dyadic model that states attachment and interpersonal dyadic processes contribute to health-related processes and understanding them is important for developing effective attachment-based health interventions. Finally, the psychotherapeutic recommendations of the meaning and attachment-based intervention (MABI; Maunder and Hunter 2004), that is an attachment-existential formulation of factors associated with medically unexplained symptoms is considered. Based on this theoretical background, the MCDT attachment-based intervention model considers possible interactions of the mother–child-disease triangles and aimed at improving the mother–child relationship in the context of the child’s disease.

MCDT was conducted using one assessment session and three separate interventions: Part A for mother, Part A′ for child, and Part B for the mother–child dyad (Table 1). The assessment session applied a comprehensive assessment of the mother–child relationship and attachment, and their general health, as well as used to establish a positive therapeutic alliance. Then, mother/child received their respective three individual sessions (parts A/A’) focused on cognition, emotion, and behavior.

**Table 1:** An overview of intervention plan included in the sessions of “mother–child-disease triangle” attachment-based intervention model

Part	Session No.	Target group	Session aims	Techniques
–	1	Mother–child dyad	Initiating therapeutic relationship; determining the mother’s and child’s thoughts, behaviors and emotions within mother–child interaction system in the context of present medical condition; outlining the goals, process and rules of the therapeutic sessions; establishing the therapeutic contract and the therapeutic alliance	Assessment interview Motivational interview
A	2	Mother	Improving mother’s recognitions about herself, her child, and the child’s disease, and these recognitions’ interaction	Dialectic and Socratic conversation/Cognitive modification techniques
	3		Improving mother’s emotions in relation to her child and child’s disease; modifying past experiences	Catharsis focused dialogues/Emotional modification techniques
	4		Improving mother’s behavioral skills and stress coping strategies, correcting the pattern of her relationship with her child and mothering behaviors	Role playing/ Psycho-educational techniques/ Practice/Homework
A′	5	Child	Improving child’s recognitions about himself, his disease, and his mother, and these recognitions’ interaction	Dialectic and Socratic conversation/Cognitive modification techniques
	6		Improving child’s emotions in relation to his mother and also his disease; modifying past experiences	Catharsis focused dialogues/Emotional modification techniques
	7		Improving child’s behavioral skills and stress coping strategies, correcting the pattern of his relationship and communication skills with his mother	Role playing/ Psycho-educational techniques/ Practice/Homework
B	8	Mother–child dyad	Mother and child reunion; observing, giving feedback, practicing and correcting mother–child relationship patterns	Psycho-educational techniques/Practice/ Role playing/Homework
	9			
	10			

The cognition session addressed the mother/child’s cognitions (beliefs) about the disease, the self and the other. A significant focus was to help reframe erroneous beliefs (faulty thinking) into healthier, reality-based structures. A goal was to help the mother/child achieve a coherent description of the medical condition and enhance their cognitive belief systems. This was done via dialectic and Socratic conversation. The emotion session focused on mediating negative emotional experiences the mother/child had internalized from previous medical events and experiences and understand how those experiences still impacted their relationship. As the researcher/therapist offered empathic attunement, the mother/child were encouraged to experience and heighten their emotions. Then, through corrective emotional-and catharsis-focused dialogues, the threatening and sometimes dissociated affects, were activated in trace form and regulated sufficiently through the process of transformation of meaning. The behavior session focused on psycho-educational techniques. The mother/child’s patterns of coping styles and relationship-building skills were targeted. This included such behavioral constructs as uninhibited reflexive responses, overly sensitive behaviors, and psychic and/or physical availability. For instance, the mother worked on her capacity for emotional attunement including ability to hear, see, understand, and respond to her child’s verbal and nonverbal cues (e.g., disease cues), and engage with her child through play, non-verbal attention, and child-directed descriptive speech to create a climate of nurturing support and secure attachment. Another example was to improve the child’s skills to benefit from mother’s support as s/he learned to make a request and express her/his needs and feelings. Finally, in Part B, the mother and child joined to test their newly learned emotional-cognitive-behavioral skills and practice how to interact with each other in the current medical condition. This part contained three sessions full of psychoeducational techniques such as role playing, practice and feedback. Additionally, the therapist encouraged joint attention and shared affect between mother and child in the context of exploratory or pretend play.

### Instruments

This study used the mother form of IPPA to appraise the quality of child’s attachment to mother. The mother’s attachment to child was assessed by mother’s attachment to child subscale of the PSI. Furthermore, GHQ-28 was used to assess the mother’s health indices, and CHQ-28 and IPQ were used to evaluate the children on health dimensions. It is important to note that the Iranian versions of these instruments were utilized in this study.

The inventory of parent and peer attachment (IPPA) is a 25-item questionnaire widely used to measure the child’s attachment to parent and peers. Studies have shown this scale’s validity and reliability with a coefficient range of 0.87–0.92 (Pace et al. 2011; Van-Ryzin and Leve 2012). This range was repeated in the Persian form of IPPA (Atashrouz et al. 2007).

The parent stress inventory (PSI) is a 101-item questionnaire that consists of two domains: the parent domain which incorporates questions about the parent’s ability to cope with the task of parenting, and the child domain which measures the parents’ perceptions of the child’s characteristics. Each domain consists of different subscales that provide detailed information about different aspects of mother and child traits. Higher scores on a subscale indicate a higher level of problems reported by the parent.

The PSI has adequate psychometric properties with a reliability coefficient of 0.93 (Deater-Deckard and Scarr 1996; Ketelaar et al. 2008). In the present study a valid Persian translation of the PSI with reliability coefficients of 0.86 and 0.83 for child and parent domains, was used. The reliability coefficient of 0.88 was reported for the total scale and retest reliability coefficient was estimated 0.94 by a 10-day time interval (Dadsetan et al. 2006). In this study, the mother’s attachment to child subscale was used.

The 28-form General Health Questionnaire (GHQ-28) is a self-administered clinical screening questionnaire originally designed for detection and assessment of individuals with an increased likelihood of a psychiatric disorder. However it is often used to detect general health indices and psychological well-being in primary care settings (Furukawa et al. 2001). It highlights physical symptoms, anxiety and sleep disorders, social disorders and severe depression subscales, and a total score for general health. Reliability coefficients range from .78 to .95 (Furukawa et al. 2001) and .84 to .91 for Iranian version (Mokhtari et al. 2013). GHQ has been used as a measures assessing parental health in this study.

The 28-form Child Health Questionnaire (CHQ-28) measures physical, social and mental health for children aged 5 years and older, and is completed by the parent. Janssens, Gorter, Ketelaar, Kramer and Holtslag (2008) introduced this instrument as a valid (Cronbach’s alpha of .62–.83) and feasible proxy measurement of child health and health related quality of life in children with a chronic medical condition. The Cronbach’s alpha of the questionnaire in the Iranian version was estimated from .70 to .85 (Ahmadi et al. 2012).

The Illness Perception Questionnaire (IPQ) is a 9-item questionnaire designed for examining cognitive representations of the illness. It measures consequences of illness, illness time-line, personal control over illness, curability, illness identity, worry about illness, illness knowledge, and affective responses to illness. It has shown good internal reliability (Cronbach alpha’s from .79 to .89) and test–retest reliability (r = .46–.88) in health-related conditions (Stockford et al. 2007). The Cronbach’s alpha in the Iranian version was estimated at .53 (Bazzazian and Besharat 2012).

## Results

Statistical analysis included the use of MANCOVA to determine whether the attachment-based intervention was effective. Here, the scores of the post-treatment indices of the IPPA, PSI, CHQ, IPQ, and GHQ served as the dependent variables, with the MCDT intervention as the independent variable and the scores of pre-treatment indices as covariates. Normality of the outcome variables were checked and there were no outliers or missing data. After examining the hypothesis of linearity, homogeneity of regression lines, and homogeneity of variances, the effect of the intervention on the dependent variables was studied.

Descriptive statistics for the pre- and post- test scores of the IPPA and the mother’s attachment subscale of the PSI in experimental and control groups are shown in Table 2. MANCOVA results are presented in Table 3, where it is apparent that the intervention produced significant changes in child’s attachment to mother (F_1,21_ = 7.25, p = .02) and mother’s attachment to child (F_1,21_ = 9.87, p = .00) in the experimental group. The Eta squared coefficients showed an effect size of 38 and 51%. It can be argued that the group factor as the independent variable caused a significant difference (up to 51%) between the experimental and control groups in mother–child attachment.

**Table 2:** Descriptive indexes for the mother–child attachment (IPPA and mother’s attachment subscale of the PSI) prior to and following treatment

Variables	Experimental				Control			
	*M*		*SD*		*M*		*SD*	
	Pre	Post	Pre	Post	Pre	Post	Pre	Post
Child’s attachment to mother	44.45	58.1	9.31	5.18	43.64	44.73	9.36	9.76
Mother’s attachment to child	18.09	14.91	3.11	1.3	17.91	18.09	2.25	2.62

**Table 3:** Results of MANCOVA for mother–child attachment (IPPA and mother’s attachment subscale of the PSI) in the experimental and control groups

Variable	*F*	*Sig*.	*η* ^*2*^
Child’s attachment to mother	7.25	.02*	.38
Mother’s attachment to child	9.87	.00**	.51

*df*=(1,99) *p < .05 **p < .01

Descriptive statistics for the experimental and control groups, considering of the pre- and post-test scores of GHQ-28, are shown in Table 4. It can be observed that both groups are equivalent according to the mothers’ health indices scores. MANCOVA results (Table 5) show that the intervention produced significant improvement in physical symptoms (*F*_1,21_ = 10.22, *p* = .00), anxiety (*F*_1,21_ = 4.52, *p* = .04), depression (*F*_1,21_ = 6.26, *p* = .02), and general health (*F*_1,21_ = 9.34, *p* = .00). The Eta squared coefficients show an effect size of .41, .34, .32, and .54 for these indices. It can be argued that the independent variable (group factor) caused a significant difference (up to 54%) between the experimental and control groups along these dimensions of health. Comparing the means show that these differences are reflected in a reduced indices score for the experimental group, meaning that the intervention decreased the intensity of health problems among mothers in experimental group.

**Table 4:** Descriptive indexes for mothers’ health (GHQ-28 subscales) prior to and following treatment

Variables	Experimental				Control			
	*M*		*SD*		*M*		*SD*	
	Pre	Post	Pre	Post	Pre	Post	Pre	Post
Physical symptom	8	2.91	3.43	2.34	8	7.27	3.4	3.95
Anxiety	9	4.55	3.4	2.46	9	7.82	3.4	4.71
Social functions	7.64	5.64	4.65	2.73	7.5	6.1	4.7	3.23
Depression	8.18	2.73	5.86	2.68	8.36	6.82	5.98	4.93
General health	8.2	3.2	3.17	1.97	8.25	7	3.66	2.98

**Table 5:** Results of MANCOVA for mothers’ health (GHQ-28 subscales)

Variable	*F*	*Sig*.	*η* ^*2*^
Physical symptom	10.22	.00**	.41
Anxiety	4.52	.04*	.34
Social functions	1.15	.74	.02
Depression	6.26	.02*	.32
General health	9.34	.00**	.54

*df*=(1,15) *p < .05 **p < .01

Table 6 includes the means and standard deviations in the experimental and control groups for the pre- and post-test scores of children’s health subscales. MANCOVA of the BDI, CHQ, and IPQ subscales shows the experimental group, as compared with the control group, to be improved on the scales of depression (*F*_1,21_ = 31.92, *p* = .000), physical health (*F*_1,21_ = 26.1, *p* = .000), social health (*F*_1,21_ = 15.61, *p* = .001), and mental health (*F*_1,21_ = 25.56, *p* = .000), but not on perception of illness (Table 7). It also shows an effect size range of .68, .63, .51, and .63 for these indices, which means that the group factor made up to 63% of the differences between the post-test means of children’s attachment dimensions in experimental and control groups, respectively. Furthermore, comparing the pre- and post-test means shows that the MCDT caused an improvement on these subscales of children’s health dimensions in the experimental group. However, no changes were observed for the dimensions of illness perception.

**Table 6:** Descriptive indexes for children’s health (BDI, CHQ, and IPQ subscales) prior to and following treatment

Variables	Experimental				Control			
	*M*		*SD*		*M*		*SD*	
	Pre	Post	Pre	Post	Pre	Post	Pre	Post
Depression	6.82	4.27	2.56	.78	6.55	9.27	3.77	2.72
Illness perception	42.27	42.82	12.08	6.81	40.09	46	13.14	4.71
Physical health	19.18	27.55	7.15	3.2	17.18	16.55	5.7	5.1
Social health	50.36	65.55	8.58	10.41	49.27	49	9.38	7.81
Mental health	69.55	93.09	14.06	11.44	66.36	65.55	12.8	10.02

**Table 7:** Results of MANCOVA for children’s health (BDI, CHQ, and IPQ subscales)

Variable	*F*	*Sig*.	*η* ^*2*^
Depression	31.92	.000***	.68
Illness perception	1.93	.18	.11
Physical health	26.1	.000***	.63
Social health	15.61	.001***	.51
Mental health	25.56	.000***	.63

*df*=(1,15) *p < .05 **p < .01 ***p < .001

## Discussion

A review of the literature suggested the necessity of using attachment as a framework for understanding and intervening in chronic medical conditions for children and their mothers (Kidd et al. 2016). One main concern was the almost total lack of experimental evidence on attachment-based intervention models specified for medical situations and children with chronic disease. From a review of attachment theories and available attachment-based models, a Mother–Child-Disease Triangle (MCDT) attachment-based intervention model was proposed. This study was designed to evaluate the notion that MCDT can improve health conditions among children with chronic disease and their mothers. The changes in the health indices of a group of children with chronic disease and their mothers were assessed before and after participating in the MDCT treatment intervention and compared with a matched group of children with chronic disease and their mothers who received a dummy intervention. The results showed significant improvement in the post-treatment stage in the MCDT intervention group in comparison with the dummy intervention group. Changes were observed in children’s depression and physical, social, and general health, and mothers’ psychical symptoms, anxiety, depression, and general health. Accordingly, it seems that MCDT could decrease the severity of these health symptoms for inpatient children and their mothers. Earlier studies also demonstrated the efficacy of attachment-based intervention models (i.e. Allen et al. 2014; Crugnola et al. 2016; Kamal et al. 2016; Moretti et al. 2015; Shpigel et al. 2012; Zilberstein and Messer 2010) but possessed limitations that reduced their usefulness in medical conditions. These attachment-based intervention models were done with families, couples or individuals of different ages and from different situations (i.e. adopted or abused children or adolescents). Though the studies were designed specifically for attachment and/or psychologically traumatic situations, none of the experimental studies included attachment-based interventions in medical conditions specifically with children and adolescents with chronic disease (Maunder and Hunter 2004;; Pietromonaco and Powers 2015). The present study, for the first time, matched previous attachment-based intervention models with special medical conditions of children suffering from chronic disease and their mothers.

Some researchers developed models that described pathways by which attachment could affect a patient’s health or disease condition. These models were the best basis and explanation for the development of this study’s MCDT attachment-based intervention model on health indices in children with chronic disease and their mothers. For instance, in the Meredith et al. (2008) model, disease, as a stressor, triggered attachment-related cognitive appraisals of disease as a threat, the self as equipped to manage the threat, and others as supportive in the situation. These appraisals impacted the behavioral responses of the person, including his/her selection of coping strategies, support-seeking behaviors, and emotional states. These attachment-related cognitive, behavioral, and emotional adaptive mechanisms demonstrated a positive increase in the quality of the person’s adjustment to her/his medical condition. Therefore, the researcher hypothesized that working on the children and mothers’ cognitions, in other words, their core beliefs of self, other and the disease, would positively impact cognitions, behavior and affect (from CBT we know that cognition drives behavior and affect). It was believed MCDT sessions could increase mental health directly and physical health indirectly through these mediating factors (e.g. coping strategies, support-seeking, adjustment, etc.). This assumption was supported by a Sadava et al. (2009) study where four mediating pathways linked attachment orientations with health/disease conditions: affect, stress, social support, health-risk behavior. In this model, as well as the direct effects of attachment on physical health, attachment was linked to physical health indirectly, through these intervening pathways (also in: Kidd et al. 2016). These mediating pathways were the main intervention contents in the MCDT model, too. One of the pathways in the Sadava et al. (2009) model was a social dimension. In this study, a social dimension also demonstrated significant improvement in children after receiving MCDT. According to Sadava et al. (2009), this improvement could help the child seek support from others (i.e. mother) and provide feelings of security in the stressful situation. In this case, the child could establish better supportive close relationships because of the attachment work as well as increase support seeking and feelings of security. Moreover, social networks are a source of tangible support in disease condition and the perception of available social support contributes to decreased stress and increased health.

The Pietromonaco et al. study (2013) is another support to explain the findings in the current study. Pietromonaco, Uchino and Schetter stated that attachment style could shape dyadic processes including relationship behaviors (e.g., support seeking, caregiving) and relationship outcomes (e.g., partner responsiveness, relationship satisfaction). These processes could influence and are influenced by physiological responses, affect, health behavior, and disease outcomes. Furthermore, partner A’s attachment style may affect partner B’s relationship mediators and outcomes and vice versa. Partner B’s health and disease outcomes could affect partner A’s physiology, affect and health behavior. Pietromonaco and Powers (2015) stated this framework highlighted opportunities for integrating theory with future intervention development in health psychology. Applying principles from Pietromonaco and colleagues to the present study model of the mother–child relationship demonstrates that an attachment-based intervention could help to correct dyadic interactions, behaviors, affects and skills in mother–child and could improve their attachment, templates of responses to medical condition and, consequently, their health. This was the main point of the MCDT, especially in Part B sessions, that it could improve children and mothers’ health by the same pathways that Pietromonaco et al. (2013) mentioned in their proposed model.

**Fig. 1:**
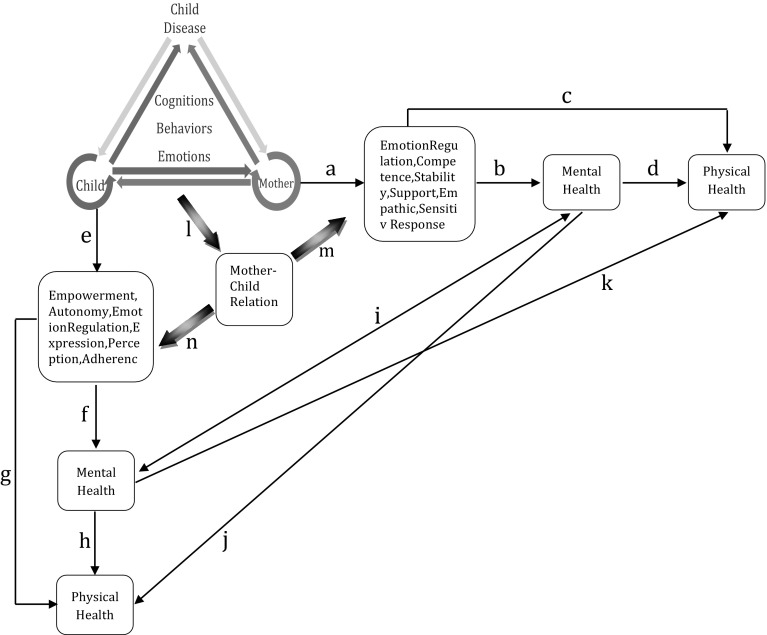
Proposed framework explaining the effectiveness of the MCDT attachment-based intervention model on health indices in children with chronic disease and their mother

Finally, regarding all above models and findings we suggested a model explaining how MCDT could improve children and mothers’ health in current study. This prototype model (Fig. 1) assumed that working on cognitive, behavioral, and emotional dimensions of the mother–child-disease interactive system during the MCDT’s mother sessions, may have improved the mother’s emotion regulation skills, competency perspective, sense of stability and security, supportive behaviors, and sensitive empathic understanding and response in relation to her child and the disease condition (path a). These changes then, may have improved the mother’s mental health (path b) and also physical health directly (path c) or indirectly through mental health (path d). The mother’s mental health improvement may have also increased the child’s mental and physical health (path i/j). The same process can be assumed for the child sessions which probably increased the child’s adaptability, sense of empowerment and autonomy, mood, emotion regulation and self-expression skills, balanced dependency, realistic perception about the disease, and therapeutic adherence (path e). These changes may have improved the child’s mental (path f) and physical health (path g/h). This improvements in the child’s health probably have had positive effects on the mother’s health too (path i/k). Furthermore, improving the mother–child relationship through the Part B sessions of the MCDT (path l) may have increased the mother and child’s mental health (path m/n).

These relationships are just assumed and the pathways outlined in the proposed model were not tested in this study. This is a proposed framework to develop and examine an attachment-based intervention model in pediatric health psychology. More experimental studies with covariance structural modeling or qualitative analyses are needed to demonstrate its validity and reliability. This current study examined the preliminary findings of the MCDT on health indices, but future studies are expected to assess the efficacy of this proposed intervention model on mother–child attachment and relationship indices, too. The moderating effects of the attachment improvement on improving health and those proposed potential pathways can be examined and discussed relative to previous study findings. The effects of the MCDT model on general physical symptoms were studied (according to CHQ and GHQ physical subscales) but not specific physical conditions particularly as they relate to children’s chronic diseases. This can be assessed in future studies.

There are limitations to this study. This was a preliminary study designed to examine a newly developed attachment-based intervention, MCDT. A small sample size was available at the hospital. With a limited number of participants, it was not possible to include all types of pediatric chronic diseases and randomly assign subjects to groups matching for the variables such as disease and sex. This could have adversely affected the research. A larger sample size and more controls are obvious necessities for future studies. The small sample size also created a lack of ethnic diversity and, therefore, findings may not be generalizable to individuals from racial/ethnic minority backgrounds. A larger number of subjects would increase observable outcomes from the intervention on a greater scale. This would enable researchers to examine the effects of intermediate variables such as personality traits of the child and mother, role of the father, disease severity, and socioeconomic condition, among others in the study. And follow-up studies are needed to assess maintenance of study results over time.

Finally, any relational engagement, especially with an empathic listener, is therapeutic by its very nature, so even though the “simple conversational sessions” for control group were classified as “dummy intervention” they still had an element of therapeutic effect which could not be controlled. Future studies could control for the effects of relational engagement if a third matched group was added with no intervention. Also delivering both the MCDT and the control intervention sessions by one therapist is another limitation of this study, because of the therapist bias risk. Although the participants in both experimental and control groups and the person who managed the pre- and post-test assessments were kept blind, it was not possible for the therapist to administer sessions without knowing which participants were receiving MCDT intervention and which were receiving dummy intervention. It also can be considered as a major concern regarding the lack of fidelity measures in this study. Arranging double-blind designs for future studies would control this fidelity effect more carefully.

This study has implications for current and future clinical practice. The results supported that considering “attachment” as an important factor in psychological interventions applied in pediatric chronic disease situations can improve the outcomes for children and their mothers. The major clinical contribution of this study is the proposed new attachment-based interventional model for clinical practitioners. Additionally, the model, being newly proposed and a relatively novel practice model, raises a number of opportunities for future research, especially in terms of model development. More research will in fact be necessary to refine and further elaborate the proposed MCDT model. More studies should be done to strongly investigate and discuss the validity, reliability and feasibility of the structure, content and techniques, before establishing the MCDT as an effective health-psychological intervention model.
